# Ecological shifts of salivary microbiota associated with metabolic-associated fatty liver disease

**DOI:** 10.3389/fcimb.2023.1131255

**Published:** 2023-02-14

**Authors:** Min Wang, Li-Ya Yan, Cai-Yun Qiao, Chu-Chu Zheng, Chen-Guang Niu, Zheng-Wei Huang, Yi-Huai Pan

**Affiliations:** ^1^ Institute of Stomatology, School and Hospital of Stomatology, Wenzhou Medical University, Wenzhou, China; ^2^ Department of Endodontics, School and Hospital of Stomatology, Wenzhou Medical University, Wenzhou, China; ^3^ Department of Endodontics, Shanghai Ninth People’s Hospital, Shanghai Jiao Tong University School of Medicine, Shanghai, China; ^4^ College of Stomatology, Shanghai Jiao Tong University, Shanghai, China; ^5^ National Clinical Research Center for Oral Diseases, National Center for Stomatology, Shanghai, China; ^6^ Shanghai Key Laboratory of Stomatology & Shanghai Research Institute of Stomatology, Shanghai, China

**Keywords:** metabolic-associated fatty liver disease, 16S rRNA sequencing, salivary microbiome, insulin resistance, diagnostic model

## Abstract

**Introduction:**

Metabolic-associated fatty liver disease (MAFLD) is the most common chronic liver disease related to metabolic syndrome. However, ecological shifts in the saliva microbiome in patients with MAFLD remain unknown. This study aimed to investigate the changes to the salivary microbial community in patients with MAFLD and explore the potential function of microbiota.

**Methods:**

Salivary microbiomes from ten MAFLD patients and ten healthy participants were analyzed by 16S rRNA amplicon sequencing and bioinformatics analysis. Body composition, plasma enzymes, hormones, and blood lipid profiles were assessed with physical examinations and laboratory tests.

**Results:**

The salivary microbiome of MAFLD patients was characterized by increased α-diversity and distinct β-diversity clustering compared with control subjects. Linear discriminant analysis effect size analysis showed a total of 44 taxa significantly differed between the two groups. Genera Neisseria, Filifactor, and Capnocytophaga were identified as differentially enriched genera for comparison of the two groups. Co-occurrence networks suggested that the salivary microbiota from MAFLD patients exhibited more intricate and robust interrelationships. The diagnostic model based on the salivary microbiome achieved a good diagnostic power with an area under the curve of 0.82(95% CI: 0.61–1). Redundancy analysis and spearman correlation analysis revealed that clinical variables related to insulin resistance and obesity were strongly associated with the microbial community. Metagenomic predictions based on Phylogenetic Investigation of Communities by Reconstruction of Unobserved States revealed that pathways related to metabolism were more prevalent in the two groups.

**Conclusions:**

Patients with MAFLD manifested ecological shifts in the salivary microbiome, and the saliva microbiome-based diagnostic model provides a promising approach for auxiliary MAFLD diagnosis.

## Introduction

Metabolic-associated fatty liver disease (MAFLD), a terminology updated from non-alcoholic fatty liver disease (NAFLD) in 2020, is the hepatic manifestation of the metabolic syndrome with a broad spectrum of liver conditions ranging from simple hepatic steatosis to steatohepatitis to stage 4 fibrosis (Demirtas and Yilmaz, 2020; Eslam et al., 2020). It usually manifests clinically silent and has no approved pharmacotherapy; with time, it can gradually progress to end-stage liver diseases, such as cirrhosis and hepatocellular carcinoma(Eslam et al., 2020). Accumulating evidence has indicated that MAFLD is strongly associated with increased risks of incident diabetes, chronic kidney disease, and cardiovascular disease, with the magnitude of risk seeming to parallel the severity of MAFLD(Liang et al., 2021; Targher et al., 2021). Globally, MAFLD affects approximately a quarter of the adult population, placing an enormous burden on healthcare systems and society(Eslam et al., 2020). Thus, convenient diagnostic screening and timely strategic intervention for MAFLD are of great significance.

The oral microbiota is the second most diverse microbial ecosystem after that of the gut in the human body, consisting of over 700 bacterial species. It plays an essential role in physiological, metabolic, and immunological functions, which include nutrient digestion, metabolic regulation, immune response, and antibacterial activity (Kilian et al., 2016). In addition to oral diseases, oral microbiota dysbiosis has also been closely linked with metabolic disorders, including MAFLD (Zhao et al., 2020; Peng et al., 2022). An animal study demonstrated that the endotoxemia from *Porphyromonas gingivalis* was a remarkable risk factor for NAFLD pathogenesis, and the altered glucose/lipid metabolism may facilitate disease progression(Sasaki et al., 2018). Our previous work has also confirmed the community structure alterations and microbial dysbiosis changes of supragingival microbiota in patients with MAFLD(Zhao et al., 2020). Given the oral cavity being a complex microbial environment, further insights into the alterations of the oral microbial community under the pathological state of MAFLD are hence warranted.

Saliva is a heterogeneous biofluid containing various proteins, metabolites, microbes, and their genes, which are essential for maintaining oral homeostasis(Belstrøm, 2020). Spreading throughout the entire oral cavity, saliva theoretically acts as a reservoir pool of microorganisms detached from various oral niches and appears representative of the overall oral microbiome(Yoshizawa et al., 2013; Belstrøm, 2020). Besides, saliva-based microbial, immunologic, and molecular biomarkers have been progressively investigated as diagnostic tools for several diseases due to the remarkable advantages of a non-invasive, stress-free, and cost-effective sampling manner(Yoshizawa et al., 2013; Zhang et al., 2016). With the rapid development of next-generation sequencing technology, a growing number of studies have evaluated the alterations of salivary microbial communities and the diagnostic utility in various diseases, such as type 2 diabetes mellitus, schizophrenia, and rheumatic heart disease, among others(Zhang et al., 2016; Liu et al., 2021; Qing et al., 2021; Shi et al., 2021). However, there is a paucity of information about salivary microbiota’s potential role in MAFLD.

This study aimed to investigate the ecological shifts in the salivary microbiome of MAFLD patients and explore the potential function of salivary microbiota. Firstly, microbiota profiles of saliva samples were characterized by 16S rRNA gene sequencing to identify changes between MAFLD patients and control subjects. Then, the potential value of the saliva-based microbiota diagnostic model was evaluated to discriminate MAFLD patients from control subjects. Finally, the relationships between clinical variables and specific microbial genera were assessed. These findings could provide more significant insights into the salivary ecological dysbiosis associated with MAFLD.

## Materials and methods

### Study population

A total of ten MAFLD patients and ten healthy control individuals were recruited from a health census. Both groups were statistically comparable in age and gender. For each subject, upper abdomen ultrasonography was performed and interpreted by experienced sonographers. MAFLD was diagnosed based on ultrasonographic findings and the exclusion of known etiologic factors of chronic liver disease as well as excessive alcohol consumption. The exclusion criteria were similar to a previous study as follows(Zhao et al., 2020):(1) excessive alcohol consumption: >30g/d for males and >20g/d for females; (2) other liver diseases, including viral hepatitis, autoimmune hepatitis, and hepatolenticular degeneration; (3) drug-induced steatohepatitis (e.g., tamoxifen, amiodarone, valproate, methotrexate, and glucocorticoids); (4) Other factors that may result in hepatic steatosis, including total parenteral nutrition, inflammatory bowel disease, celiac disease, hypothyroidism, Cushing’s syndrome, lipoprotein deficiency, lipid-atrophic diabetes, etc.; (5) type I or type II diabetes; (6) the use of lipid-lowering medication within the past six months; (7) other conditions, including pregnant or lactating women, prolonged heavy smoking, use of antibiotics for more than five days within six months, etc.; (8) untreated oral abscess, oral precancerous lesions and oral cancer, oral fungal infections; more than eight teeth missing. This study followed the Declaration of Helsinki on medical protocols and ethics, and was approved by the Ethics Committee of School and Stomatology Wenzhou Medical University (approval no. WYKQ2021006). Written informed consent was obtained from all participants.

### Anthropometric and clinical variables measurement

All participants underwent physical examinations, oral examinations, and anthropometric measurements, together with fasting blood sample collection for biochemical tests. The anthropometric parameters included body weight, height, body mass index (BMI), waist and hip circumferences, waist-hip ratio (WHR), blood pressure, and heart rate. The blood biochemical indicators included total cholesterol (TC), total triglycerides (TG), low-density lipoprotein cholesterol (LDL-C), high-density lipoprotein cholesterol (HDL-C), alanine aminotransferase (ALT), aspartate aminotransferase (AST), gamma glutamyl transpeptidase (GGT), fasting plasma glucose (FPG), fasting serum insulin (FSI), glycosylated hemoglobin (HbA1c) and C-reactive protein (CRP). In addition, the Homeostatic Model Assessment for Insulin Resistance (HOMA-IR) was used to ascertain IR. The formula is as follow: HOMA-IR = FPG (mmol/L) × FSI (mU/L)/22.5(Matthews et al., 1985). A thorough dental exam was conducted to assess dental caries, periodontal status, and other dental conditions listed above by the same dentist. Interdental clinical attachment loss presents at ≥ 2 non-adjacent teeth, or buccal or oral clinical attachment loss ≥ 3 mm with pocketing >3 mm presents at ≥ 2 teeth are diagnosed as periodontitis(Tonetti et al., 2018). The number of decayed, missing, and filled teeth (DMFT) for each subject were recorded. The unpaired Student’s t-test was carried out to analyze anthropometric and biochemical indicators, except for sex and periodontitis, for which the chi-square test was used.

### Specimen collection

Unstimulated saliva samples were collected from each subject according to the Human Microbiome Project Core Sampling Protocol A (https://www.hmpdacc.org/hmp/doc/HMP_MOP_Version12_0_072910.pdf) with minor adjustments. Prior to salivary microbiota sampling, all participants were requested to avoid oral hygiene procedures for 24 h, abstain from drinking, eating at least 2 h in advance. At least 2 ml of unstimulated saliva samples were collected in sterile tubes between 9 am and 11 am. All samples were transported at 4°C to the laboratory as soon as possible and then stored in liquid nitrogen until DNA extraction.

### DNA extraction, amplification, and high-throughput sequencing

The total microbial community DNA was extracted by QIAamp DNA Mini Kit (Qiagen, Valencia, CA, USA) according to the manufacturer’s instructions, and the quality was determined using a NanoDrop 2000 UV-vis spectrophotometer (Thermo Scientific, Wilmington, DE, USA) and 1% agarose gel electrophoresis. The V3-V4 hypervariable region of bacterial 16S rRNA was amplified by a thermocycler PCR system (GeneAmp 9700; Applied Biosystems, Carlsbad, CA, USA) with the forward primer 338F (5′-ACTCCTACGGGAGGCAGCAG-3′) and the reverse primer 806R (5′-GGACTACH VGGGTWTCTAAT-3′). The PCR products were purified using the AxyPrep DNA Gel Extraction Kit (Axygen Biosciences, Union City, CA, USA) and quantified using the QuantiFluor ^®^ Single-Tube Fluorometer (Promega Corporation, Madison, WI, USA), following the manufacturer’s instructions. Finally, the purified amplicons were paired-end sequenced on an Illumina Miseq PE300 platform (Illumina, San Diego, CA, USA). The sequencing work was completed by Majorbio (Shanghai, China).

### Data processing and bioinformatics analysis

The raw sequence reads were quality-filtered using Fastp (version 0.19.6, https://github.com/OpenGene/fastp) and merged by FLASH software (version 1.2.11; https://ccb.jhu.edu/software/FLASH/index.shtml ). The specific criteria are consistent with the previous study(Zhao et al., 2020). UPARSE (version 7.1, http://drive5.com/uparse/) was used to cluster the sequences into operational taxonomic units (OTUs) with a threshold of 97% similarity. The taxonomy assignment of each OTU was carried out by RDP Classifier (version 2.11, https://github.com/OpenGene/fastp) against the SILVA 16S rRNA database (Release 132, https://www.arb-silva.de/ ) using a confidence threshold of 0.7. Prior to further analysis, the OTUs tables were subsampled to equal depths according to the fewest sample sequence.

Alpha diversity indexes (Shannon, Simpson, Ace, and Chao1) based on the OTUs profiles were applied to analyze microbial diversity, which were calculated with Mothur software (version 1.30.2, http://www.mothur.org/). The principal coordinates analysis (PCoA) and partial least squares discriminant analysis (PLS-DA) plots based on Euclidean distance at the genus level were performed to visualize the differences in species composition between the MAFLD and control groups. The species relative abundance was visualized by bar plots at the phylum and genus levels. Besides, the differences in relative abundance were analyzed at the genus level by Analysis of Composition of Microbiomes (ANCOM) *via* ANCOM 2.0 package in R platform. Statistical significance was defined as W>0.7.

The linear discriminant analysis (LDA) combined effect size (LEfSe; http://huttenhower.sph.harvard.edu/galaxy) was employed to identify the significantly different taxa between the groups at the phylum to genus levels. The threshold on the logarithmic LDA score for discriminative features was set to 2.0. Co-occurrence networks at the genus level were built by pairwise correlation spearman analysis (Spearman’s coefficient > 0.5 and P-value < 0.05). For each network, two properties named “average shortest path length” and “transitivity” were computed using NetworkX86 (version 2.4) according to a previous study(Loftus et al., 2021). A random forest classifier was trained to discriminate the MAFLD patients from healthy control subjects based on the genus abundance profile, and its prediction accuracy performance was accessed by the area under the ROC curve (AUC). (randomForest package in R V.4.3.2 and plotROC package in R V.3.4.4). The potential relationships between microbial population distribution and clinical variables were evaluated through redundancy analysis (RDA). Moreover, Spearman correlation coefficients were carried out to analyze correlations between clinical variables and the top 20 abundant genera, and the results were visualized by heat maps using the R platform. Furthermore, the functional profiles of microbial communities were predicted using PICRUSt2(version 2.2.0; http://picrust.github.io/picrust/) with reference to the Kyoto Encyclopedia of Genes and Genomes (KEGG) database. Statistical differences between the two groups were determined by Wilcoxon rank-sum test with Benjamini-Hochberg false discovery rate (FDR) correction. Statistical significance was set to *P* < 0.05

## Results

### Subject characteristics and clinical data

The demographic data, clinical characteristics, and laboratory data of the participants were summarized in **Table 1**. No statistical difference was observed in age, gender, blood pressure, heart rate, periodontitis, and DMFT between the two groups, whereas BMI and WHR were higher in the MAFLD group compared to the control group. With regard to biochemical indicators, FPG, FSI, HOMA-IR, and HbA1C were significantly elevated in the MAFLD group, suggesting the presence of IR in MAFLD patients. In addition, the MAFLD group had substantially higher TG levels and lower HDL-C levels, resulting in a higher TG/HDL-C ratio than the control group. Although there was no statistical difference between ALT and AST levels, the AST/ALT ratio was significantly reduced in the MAFLD group.

**Table 1:** Demographic and clinical characteristics of the study population.

Parameter	Healthy (n=10)	MAFLD (n=10)	*P*
Age (y)	33.30 ± 5.12	35.80 ± 9.11	0.459
Gender (male/female)	6/4	9/1	0.303
BMI (kg/cm^2^)	22.07 ± 1.24	26.33 ± 2.68	<0.001^*^
WHR	0.84 ± 0.07	0.91 ± 0.05	0.019^*^
SBP (mm HG)	118.30 ± 14.08	123.30 ± 13.40	0.427
DBP (mm HG)	75.10 ± 9.59	79.50 ± 7.28	0.263
HR	74.80 ± 5.87	79.80 ± 7.13	0.104
TC (mmol/L)	4.81 ± 0.84	4.66 ± 1.09	0.740
TG (mmol/L)	0.89 ± 0.33	1.84 ± 1.23	0.039^*^
LDL-C (mmol/L)	3.00 ± 0.95	2.94 ± 0.98	0.892
HDL-C (mmol/L)	1.44 ± 0.39	1.02 ± 0.16	0.005^*^
ALT (U/L)	32.60 ± 39.83	55.40 ± 30.35	0.167
AST (U/L)	26.50 ± 14.37	29.50 ± 11.30	0.610
AST/ALT	1.21 ± 0.55	0.63 ± 0.22	<0.001^*^
GGT (U/L)	24.00 ± 19.55	43.30 ± 24.32	0.066
FPG (mmol/L)	4.43 ± 0.46	5.26 ± 0.40	<0.001^*^
FSI (mU/L)	7.71 ± 3.02	14.80 ± 4.89	0.001^*^
HOMA-IR	1.49 ± 0.52	3.44 ± 1.11	<0.001^*^
HbA1C	5.00 ± 0.23	5.46 ± 0.29	0.001^*^
CRP	0.76 ± 1.01	1.15 ± 1.11	0.421
Periodontitis (%)	10.00	20.00	0.531
DMFT	4.50 ± 1.35	5.40 ± 2.59	0.343

Data are presented as mean ± SDs. ^*^P < 0.05. BMI, body mass index; WHR, waist-hip ratio; SBP, systolic blood pressure; DBP, diastolic blood pressure; HR, heart rate; TC, total cholesterol; TG, total triglycerides; HDL-C, high-density lipoprotein cholesterol; LDL-C, low-density lipoprotein cholesterol; ALT, alanine aminotransferase; AST, aspartate aminotransferase; GGT, gamma glutamyl trans-peptidase; FPG, fasting plasma glucose; FSI, fasting serum insulin; HOMA-IR, homeostatic model assessment of insulin resistance [HOMA-IR = FPG (mmol/L) × FSI (mU/L)/22.5]; HbA1c, glycosylated hemoglobin; CRP, C-reactive protein; DMFT, decayed, missing, and filled teeth.

### Community structure and microbiota composition of salivary microbiota

**Figure 1:**
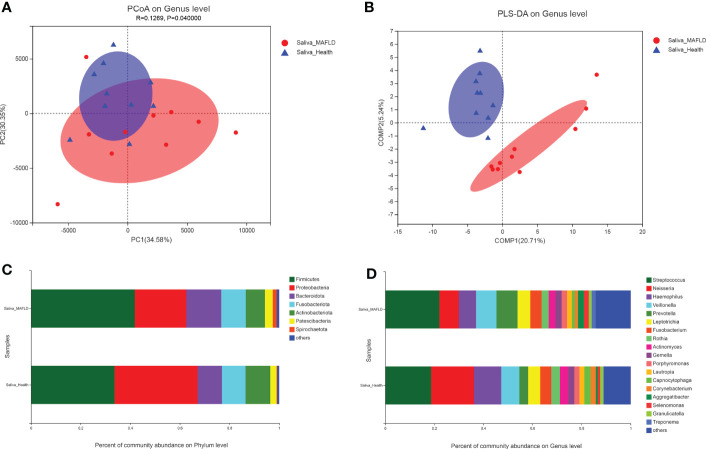
Comparison of salivary microbiota structures in the metabolic-associated fatty liver disease (MAFLD) and control groups. **(A)** Euclidean principal coordinates analysis (PCoA) at the genus level. **(B)** Partial least squares discriminant analysis (PLS-DA) at the genus level. **(C)** Microbial community structures at the phylum level. **(D)** Microbial community structures at the genus level.

Twenty saliva samples contained 16 phyla, 24 classes, 62 orders, 100 families, 205 genera, 383 species, and 453 OTUs. In terms of α-diversity, as shown in **Table 2**, the Ace and Chao1 richness index of the MAFLD group significantly increased compared with those in the control group, indicating that MAFLD considerably altered the diversity and richness of the salivary microbiota. Principal coordinate analysis (PCoA) based on Euclidean distance at the genus level was employed to evaluate the β-diversity of the salivary microbiota community, despite partial overlapping, which showed a significant difference between the two groups (AMOSIM, *P=0.04*; **Figure 1A**). The visualization of sample clustering using a supervised PLS-DA plot further confirmed the remarkable separation between groups (**Figure 1B**).

**Table 2:** α -diversity of salivary microbiota in the MAFLD and Control groups.

	Health	MALFD	*P*
Shannon	3.22 ± 0.33	3.51 ± 0.61	0.4064
Simpson	0.08 ± 0.03	0.09 ± 0.06	0.9336
Ace	224.07 ± 18.93	262.47 ± 51.40	0.03972^*^
Chao1	228.27 ± 17.19	266.97 ± 49.76	0.03202^*^

Results are presented as the mean ± SDs. ^*^P < 0.05. All α-diversity estimators were analyzed using the Student’s t- test.

The most abundant phyla in both groups were *Firmicutes, Proteobacteria, Bacteroidota, Fusobacteriota, Actinobacteriota, Patescibacteria*, and *Spirochaetota*, accounting for over 99% of community abundance. Notably, the relative abundance of *Firmicutes* increased in comparison to the controls, while the relative abundance of *Proteobacteria* declined in the MAFLD patients, thereby resulting in a lower *Firmicutes/Proteobacteria* ratio in the MAFLD group (**Figure 1C**). At the genus level, the predominant bacteria included *Streptococcus* with a relative abundance of 22.09% and 18.70%, and Neisseria with a relative abundance of 7.89% and 17.44% in the control and MAFLD groups, respectively. (**Figure 1D**).

### Alterations of the salivary microbial taxa

**Figure 2:**
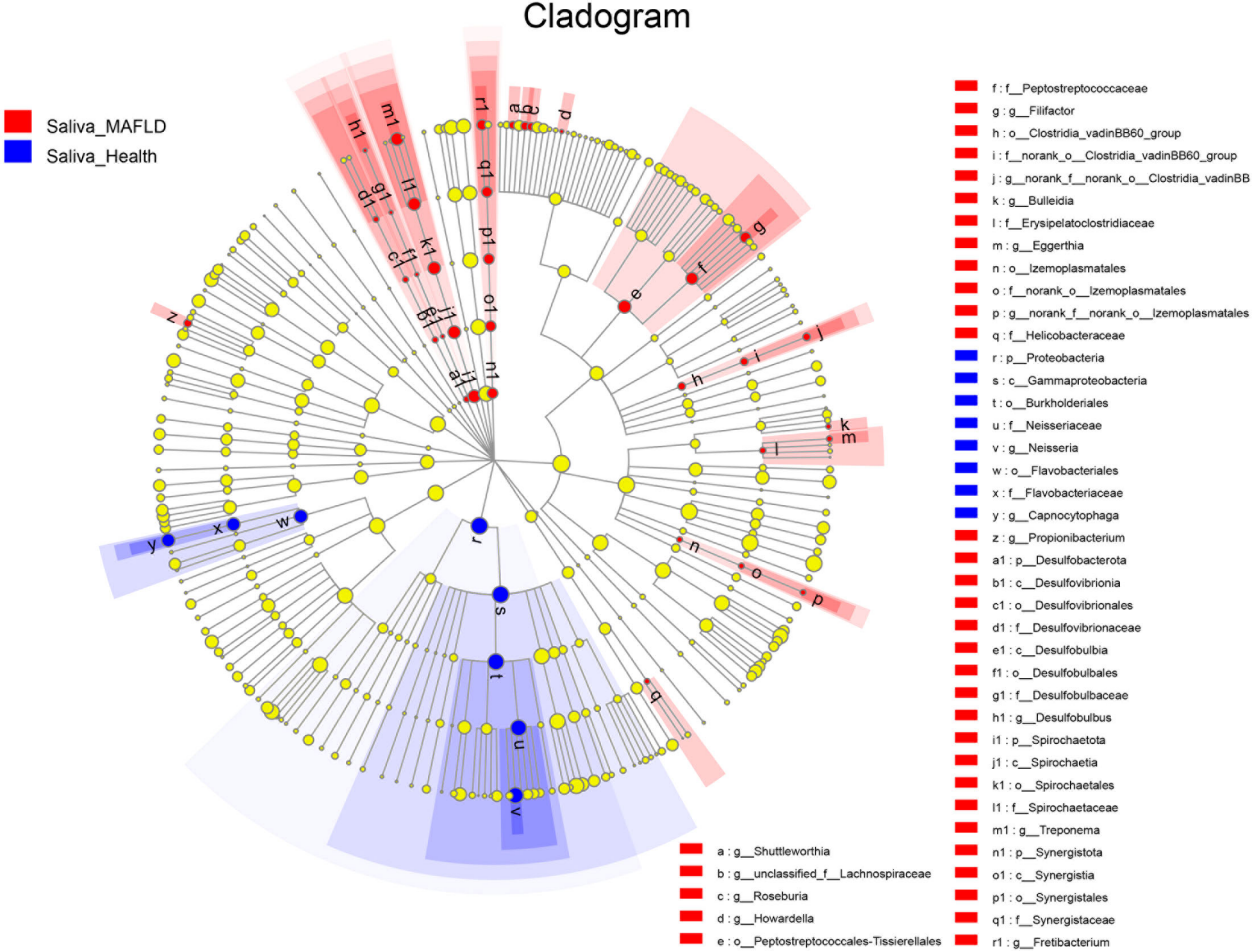
Discriminative salivary microbiota with abundance difference in the MAFLD and control groups. A cladogram of taxonomic representation based on LEfSe. Red indicates enrichment in samples from the MAFLD group, and blue indicates the taxa enriched in samples from the control group (LDA>2.0, *P*<0.05).

The genus abundance was compared based on community abundance data to assess the specific alterations in salivary microbiota. ANCOM analysis showed that the genera *Filifactor, Neisseria*, and *Capnocytophaga* had significantly different abundances between the two groups (**Table 3**). LEfSe analysis revealed that the MAFLD group had a higher abundance of genera *Howardella, Treponema, Desulfobulbus, Bulleidia, Propionibacterium, Filifactor, Eggerthia, Fretibacterium, Shuttleworthia*, and *Roseburia*. By contrast, the control subjects had a higher abundance of genera *Neisseria* and *Capnocytophaga.* (**Table 3**). **Figure 2** depicted the differentially abundant microbial taxa from phylum to genus level with LDA scores higher than 2.0 based on the LEfSe analysis. Overall, 4 phyla, 5 classes, 9 orders, 11 families, and 15 genera were identified to be significantly discriminant, with thirty-six taxa enriched in the MAFLD group and eight taxa enriched in the control group. These results suggested the presence of specific bacteria in the saliva of MAFLD patients and healthy subjects.

**Table 3:** Significantly different genus between the MAFLD and Control groups.

Taxonomic Assignment	W^a^	Group^b^	LDA Value^c^
g:Howardella	ns	MAFLD	4.05
g:Treponema	ns	MAFLD	3.84
g:Desulfobulbus	ns	MAFLD	3.65
g:Bulleidia	ns	MAFLD	3.65
g:Propionibacterium	ns	MAFLD	3.40
g:Filifactor	112	MAFLD	3.34
g:Eggerthia	ns	MAFLD	3.29
g:Fretibacterium	ns	MAFLD	3.18
g:Shuttleworthia	ns	MAFLD	2.98
g:Roseburia	ns	MAFLD	2.73
g:Neisseria	149	Health	4.68
g:Capnocytophaga	142	Health	3.94

^a^W by ANCOM statistic. ^b^The group showing high abundance by LEfSe analysis. ^c^LDA value by LEfSe analysis. ns, not significant W statistics(<W_0.7_).

**Figure 3:**
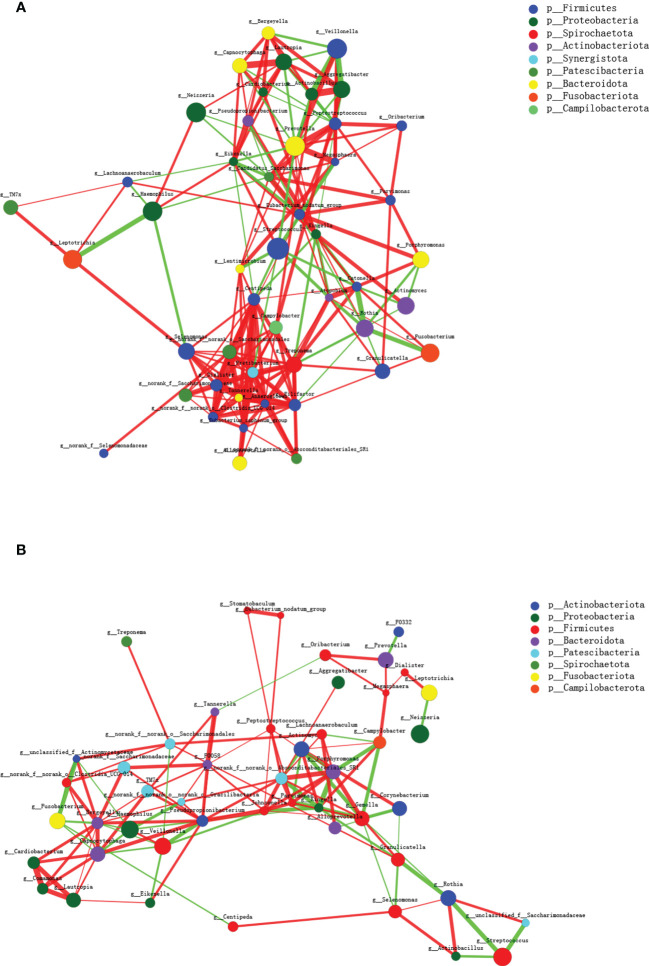
Microbial co-occurrence networks of the top 50 abundant genera constructed on the MAFLD **(A)** and control **(B)** groups. Each node represents a genus, and the size of the node is proportional to the mean relative abundance. The same color represents the genera belonging to the same phylum. The thickness of each connection is proportional to the coefficient values. The red and green lines indicate positive and negative interactions, respectively.

Co-occurrence networks among the top 50 abundant genus-level taxa were conducted further to investigate the biological interactions within the microbial community. As shown in **Figure 3**, the microbial networks slightly differ between the two groups, and the taxa within the MAFLD groups exhibited more intricate and robust interrelationships. The network transitivity for the MAFLD and health groups were 0.522 and 0.438, respectively. In contrast, the average shortest path length was 2.240 for the MAFLD group and 3.207 for the health group.

### Diagnostic model for MAFLD based on salivary microbiota

**Figure 4:**
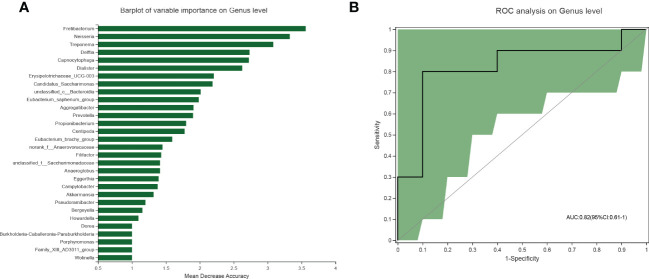
Diagnostic model based on the salivary microbiome for MAFLD. **(A)** Barplot of the genera ranked by importance in the random forest model. **(B)** The receiver operating characteristic curve achieved an AUC value of 0.82 on the genus level. And the green shade was a confidence interval.

Random forest analysis was conducted to identify the diagnostic potential of salivary microbiota for MAFLD. It is generally accepted that when the value of AUC is greater, the biomarkers have higher diagnostic accuracy. Among salivary microbiota, a panel of seven genera, including *Fretibacterium, Neisseria, Treponema, Delftia, Capnocytophaga, Dialister, and Erysipelotrichaceae_UCG-003* were identified as optimal biomarkers with an AUC of 0.82(95% CI: 0.61–1) (**Figure 4**). These outcomes indicated that salivary microbial markers achieved a good diagnostic potential for discriminating MAFLD patients from the healthy cohort.

### Correlation between clinical variables and microbial communities

**Figure 5:**
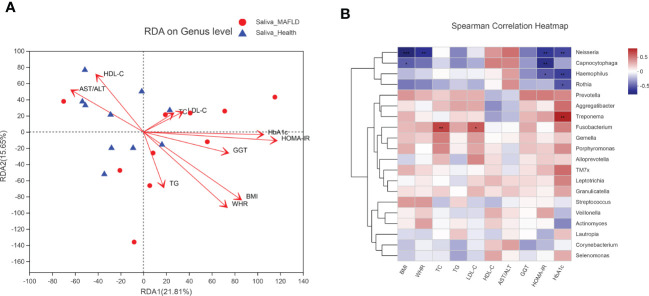
Correlation analysis between microbial community and clinical variables. **(A)** RDA analysis on microbial population distribution is explained by the clinical variables at the genus level. Arrows represent clinical variables. The long arrow indicates a high correlation with the distribution of the salivary microbiome. The acute angle of the two arrow lines indicates a positive correlation between the clinical variables, and the obtuse angle is a negative correlation. **(B)** Spearman correlation heatmap based on the top 20 abundant salivary microbiota and clinical variables. The color range on the right shows the color partitioning of the different R values. A clustering tree for each species is on the left side of the heat map. *P < 0.05; ** P < 0.01; *** P < 0.001.

To explore the potential associations between microbial community composition and multiple clinical variables, the following ten main clinical variables were chosen for the RDA test: BMI, WHR, TC, TG, LDL-C, HDL-C, AST/ALT, GGT, HOMA-IR, and HbA1c. As depicted in **Figure 5A**, HOMA-IR (r^2 =^ 0.4896, *p* = 0.007), BMI (r^2 =^ 0.4819, *p* = 0.0.003), WHR (r^2 =^ 0.4595, *p* = 0.006) and HbA1c (r^2 =^ 0.3874, *p* = 0.018) played significant roles in the salivary community composition. Furthermore, the Spearman correlation heatmap portrayed the association between the top 20 abundant genera and clinical variables (**Figure 5B**).

### Alterations of functional pathway

**Figure 6:**
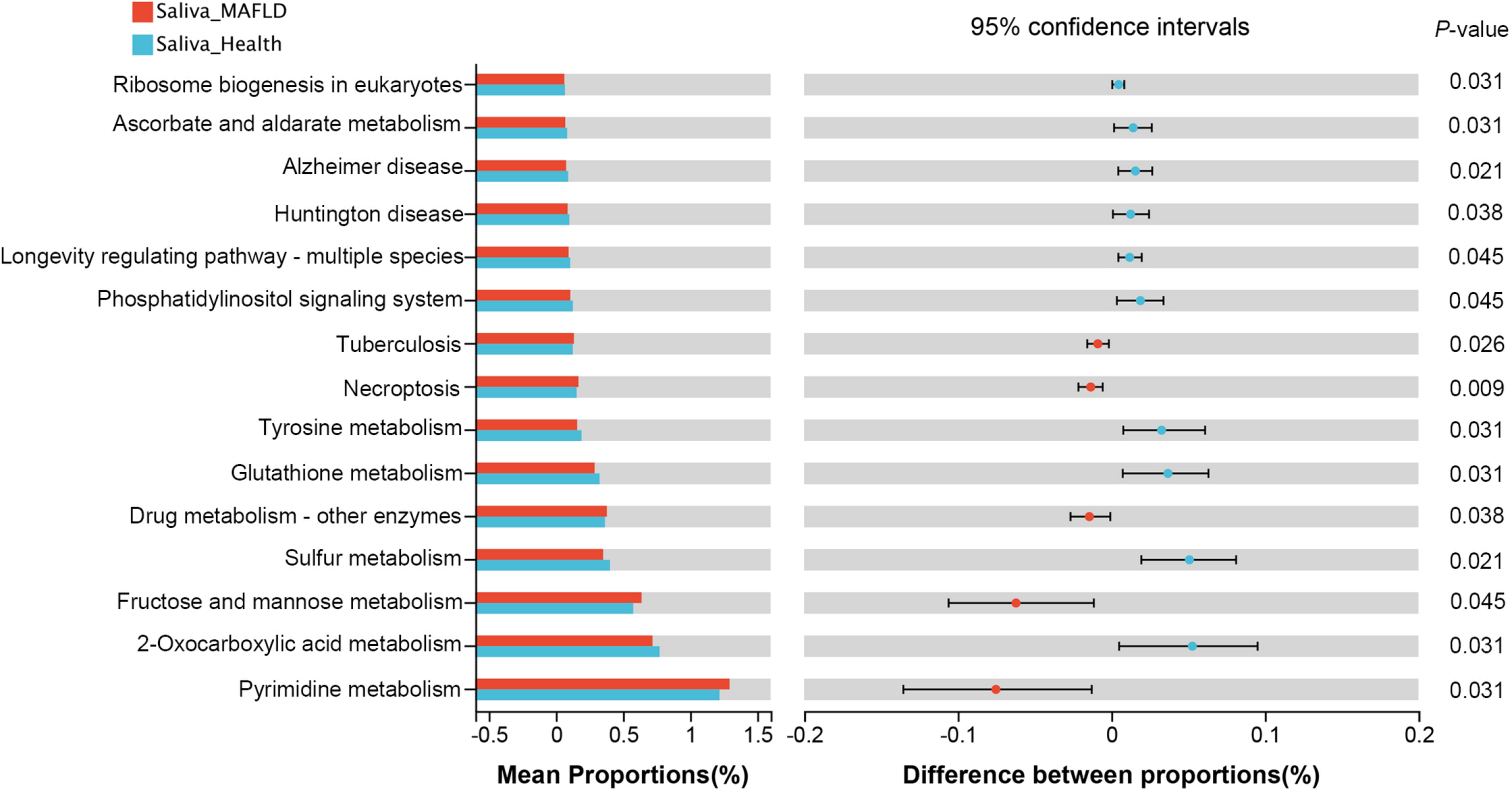
Predictive metagenome functional profiling of the top 15 significantly abundant KEGG pathways using PICRUSt2 analysis. Bar plots on the left side show the mean proportion of each KEGG pathway; on the right display the differences between proportions.

To investigate the functional implications of salivary microbiota, PICRUSt2 was performed to predict the metagenome functional content from 16S rRNA data. Notably, among the top 15 significantly abundant KEGG pathways, seven pathways belonging to the metabolism category were observed (**Figure 6**). MAFLD group exhibited a significant increase in pathways associated with pyrimidine metabolism and fructose and mannose metabolism, whereas a decrease in pathways related to 2-Oxocarboxylic acid metabolism, sulfur metabolism, glutathione metabolism, tyrosine metabolism, and ascorbate and aldarate metabolism, etc.

## Discussion

The salivary microbiota, which serves as a reservoir of microorganisms from different sites within the oral cavity, could represent the whole oral microbiota and reflect the oral and general health status(Belstrøm, 2020). An increasing number of metabolic disorders have been reported in potential relation to the shifts in salivary microbial ecology, such as atherosclerosis, type 2 diabetes mellitus, polycystic ovary syndrome, and hepatic encephalopathy (Yoshizawa et al., 2013; Zhang et al., 2016; Liu et al., 2021). However, to the best of our knowledge, few studies have explored the microbiome-level association between salivary microbiota and MAFLD. This study discovered that abnormal metabolic levels in MAFLD patients had significantly altered the composition and structure of salivary microbiota, which had good diagnostic power in discriminating MAFLD patients from healthy controls.

Oral microbial diversity is essential for maintaining health and varies with the physiological state of the host. Generally, elevated microbiome richness and diversity have been recognized as hallmarks of a healthy ecosystem, especially for the gut microbial ecosystem(Falony et al., 2018). However, our present study showed that the salivary microbiota in MAFLD patients exhibited increased α-diversity (ACE index and Chao1 index). Although no significant differences were observed in the Shannon and Simpson indices, both showed marginally elevated diversity among patients with MAFLD. These findings are consistent with our previous study that focused on the shifts in supragingival microbiota associated with MAFLD(Zhao et al., 2020). Following the concept proposed by Takeshita et al. (Takeshita et al., 2016), poor oral health could lead to an increased taxonomic richness in saliva. Because prolonged plaque accumulation may cause the multiplication of attached bacteria, and the gingival bleeding could provide rich nutrients for bacteria growth. These ecological shifts may promote bacterial assemblage in saliva. This phenomenon has been previously observed in patients with other metabolic diseases(Si et al., 2017). Furthermore, the PCoA and PLS-DA results revealed a significant clustering of microbial communities between the two groups. Thus, the altered metabolic level in patients with MAFLD would provoke salivary microbial shifts and increase the risk of oral diseases.

Phyla *Firmicutes* and *Proteobacteria* showed divergent abundance trends, with *Firmicutes* enrichment and *Proteobacteria* depletion from the control group to the MAFLD group, resulting in an increase in *Firmicutes*/*Proteobacteria* ratio. The increased *Firmicutes*/*Proteobacteria* ratio was also detected in the salivary microbiome of patients with primary Sjögren’s syndrome(Van der Meulen et al., 2018) and schizophrenia(Qing et al., 2021). Similar to MAFLD, these two diseases are characterized by chronic low-grade inflammation. Lau et al. also revealed a shift in gut microbiome toward decreased *Firmicutes*/*Proteobacteria* ratio in type 2 diabetic adults with mild obesity following metabolic surgery (Lau et al., 2021). A probable reason is that *Proteobacteria* are a major phylum of Gram-negative bacteria, mainly associated with glucose homeostasis improvement, metabolic amelioration, and inflammatory response reduction(Carvalho et al., 2012; Lau et al., 2021). In contrast, the phylum *Firmicutes* are mostly Gram-positive bacteria playing a pivotal role in the fermentation and metabolism of carbohydrates and lipids through chain-breaking fatty acid synthesis, which usually facilitates obesity development(Stojanov et al., 2020). Therefore, it is indicative that *Firmicutes* bacteria could have a competitive advantage over *Proteobacteria* bacteria in niche occupancy during the MAFLD state. However, whether the *Firmicutes/Proteobacteria* ratio could be used as a marker of ecosystem health in microbiome research still requires more studies to confirm.

LEfSe and ANCOM analyses revealed notable discrepancies between the two groups. Genus *Filifactor* was enriched in MAFLD patients, whereas genera *Neisseria* and *Capnocytophaga* were more abundant in healthy controls. Of the remaining genera, *Treponema, Fretibacterium, Propionibacterium, Shuttleworthia, Eggerthia, Bulleidia, Howardella*, and *Desulfobulbus*, presented a higher proportion in the MAFLD group. *Filifactor* spp. are well-known periodontal pathogens (Takeshita et al., 2016) and have been reported to exist in high abundance in the salivary microbiota of patients with hepatocellular carcinoma (Park et al., 2021). The genus *Treponema* consists of a diverse group of pathogenic or commensal microorganisms that not only cause syphilis, yaws, and pinta infections(Shukla et al., 2022) but are also associated with chronic liver disease(Ling et al., 2015), infective endocarditis(Hijikata et al., 2019), and Alzheimer’s disease (Riviere et al., 2002). Besides, oral *Treponema denticola*, together with *Porphyromonas gingivalis* and *Tannerella forsythia*, are known as the “red complex” which have been widely considered essential pathogens in periodontal disease etiology and pathogenesis. A series of animal and human studies have confirmed that the genera *Fretibacterium* (Yamamoto et al., 2021), *Propionibacterium* (Del Chierico et al., 2017), *Shuttleworthia* (Xie et al., 2016), *Eggerthia* (Yamamoto et al., 2021), and *Bulleidia* (Bashiardes et al., 2016)are prevalent in the oral or gut microbiota with liver disease including nonalcoholic fatty liver disease, hepatocellular carcinoma. These findings could be interpreted by the emerging oral-gut-liver axis that oral microbes could translocate into the gastrointestinal tract, where they would spread to the liver and induce hepatic diseases(Imai et al., 2021). On the other hand, *Neisseria* spp. (Liu et al., 2015) are generally Gram-negative microorganisms comprising mainly non-pathogenic species in the oral cavity. Previously, we also demonstrated a higher abundance of *Neisseria* in the supragingival plaque among healthy individuals compared to those with MAFLD (Zhao et al., 2020). *Capnocytophaga* is a genus of Gram-negative anaerobic bacteria reportedly linked with improved oral health and reduced caries experience (Schoilew et al., 2019). Interestingly, the genus *Neisseria and Capnocytophaga* have been identified as part of human oral health’s “core microbiome” (Zaura et al., 2009).

Microorganisms coexist in intricate networks of interactions, which in turn affect the species involved and may lead to disease progression. As shown in the co-occurrence networks diagram, the inter-genera interactions in MAFLD saliva exhibit higher network transitivity and lower average shortest path lengths. Transitivity measures the average connectedness of a network, with higher values indicating the presence of more tightly connected clusters (more inter-genus interactions) (Sisk-Hackworth et al., 2021). By contrast, the lower average shortest path lengths within microbial networks suggest species are interconnected through shorter paths (Loftus et al., 2021). The underlying reason may be that short path lengths and intimate connections within the salivary networks in patients with MAFLD could transmit signals rapidly between bacterial species, thereby potentially promoting shifts in community metabolism.

A timely and accurate diagnosis of MAFLD is a prerequisite for expeditious therapeutic interventions, which could inhibit disease progression. The random forest analysis showed that the salivary microbiota signature has good diagnostic power (bacterial taxa achieved an AUC of 0.82) to discriminate MAFLD patients from healthy controls. These findings reinforce the notion that saliva microbes potentially rich in diagnostic biomarkers could serve as indicators of both oral and systemic diseases(Yoshizawa et al., 2013; Zhang et al., 2016). Using 16S rRNA sequencing of fecal samples from children, Schwimmer et al. reported that prediction models combining serum ALT levels and relative abundance of encoding genes could discriminate participants in NAFLD, NASH, and severe fibrosis with AUCs of 0.95, 0,92, and 0.87, respectively(Schwimmer et al., 2019). Although gut microbiota prediction seems superior for MAFLD, given concerns about noninvasiveness and accessibility, the biochemical blood indices were not incorporated into the saliva-based biomarker model in this study. The key next step is to generalize and transform these critical microbial biomarkers into available tools for clinical practice(Mouzaki and Loomba, 2020).

With numerous studies reporting the complex etiology and pathogenesis of MAFLD, it has become clear that underlying risk factors for MAFLD development encompass IR, obesity, and dyslipidemia(Duseja and Chalasani, 2013), among which IR takes center stage (Kuraji et al., 2021). IR means the cells in the body muscles, fat, and liver cannot respond well to the normal concentration of insulin hormone that promotes glucose uptake and fat storage. Therefore, IR caused by the imbalance between energy intake and expenditure could lead to fat accumulation in the liver and promote dyslipidemia through increased circulating free fatty acids in blood(Kuraji et al., 2021). As first described by Matthews et al. in 1985, HOMA-IR has been commonly used for IR estimation in clinical studies, with a HOMA-IR value ≥2.5 indicating IR(Matthews et al., 1985).In this research, HOMA-IR in the MAFLD group exceeded the threshold value and was significantly higher than in the control group, suggesting that IR is highly prevalent among patients with MAFLD. Moreover, the elevated HbA1c and TG/HDL-C ratio, known as a simple and reliable marker of IR, further demonstrated the propensity for IR in the MAFLD group(Borai et al., 2011; Fan et al., 2019). In addition, elevated serum transaminase levels and a decreased AST/ALT ratio are markers of ongoing hepatocellular injury. They are commonly deranged in patients with progressive MAFLD(Sheka et al., 2020), implying that enrolled MAFLD patients had different degrees of hepatocellular damage.

The RDA analyses further verified the significant influences of IR (*p* = 0.007 for HOMA-IR; *p* = 0.018 for HbA1c) and obesity (*p* = 0.003 for BMI; *p* = 0.006 for WHR) over the salivary microbiota distribution. As shown in the spearman correlation heatmap (between the top 20 abundant genera and clinical variables), although some correlations did not reach statistical significance, *Neisseria*, *Capnocytophaga*, *Haemophilus*, and *Rothia* tended to be negatively associated with IR, obesity, and dyslipidemia index. These genera are members of nitrate-reducing bacteria residing in the oral cavity and exerting beneficial effects on hosts through the nitrate–nitrite–nitric oxide pathway, such as regulating glucose metabolism, lowering lipid levels, and reducing inflammation(Rosier et al., 2022). However, the specific role and related mechanisms of these oral nitrate-reducing microbes in MAFLD remain to be further delineated. The negative association results support the presumption that metabolic abnormalities (e.g., dyslipidemia, IR, and obesity) may play a role in modulating oral microbial composition and cause oral dysbiosis(Negrini et al., 2021). Genus *Fusobacterium* was positively correlated with TC and LDL-C levels, consistent with the concept that the presence of *Fusobacterium* spp. is implicated in the pathogenesis of atherosclerosis *via* aberrant lipid metabolism(Zhou et al., 2022).

The analysis with PICRUSt2 demonstrated that the main functional alterations between the two groups were metabolic-related pathways. The upregulation of the pathways for pyrimidine metabolism and fructose and mannose metabolism is consistent with our previous supragingival plaque study on MAFLD patients(Zhao et al., 2020), further confirming the metabolic-related pathways of oral bacteria are associated with the occurrence of MAFLD. Pathways related to amino acid metabolism (including glutathione metabolism and tyrosine metabolism) were more prevalent in healthy subjects, possibly because the MAFLD patients have disturbances in amino acid metabolism caused by hepatocellular injury. However, further metabolome studies are needed to investigate the metabolism in MAFLD and its relationship to the salivary microbiome.

A limitation of this study was the limited sample size. Although the inclusion criteria were rigorous, future investigations with a larger sample size were needed to control for potential confounding factors. Moreover, due to the limitation of 16s rRNA sequencing, metagenomics sequencing may be required to genetically annotate and validate functional information related to salivary microbiome changes in patients with MAFLD.

In conclusion, we comprehensively described salivary microbiome alterations between MAFLD patients and healthy individuals, evaluated the potential value of salivary microbiota as an auxiliary diagnostic tool to predict MAFLD, and demonstrated the role of biochemical variables, especially the IR, in microbial ecological shifts. This study provides an in-depth view of the association between the salivary microbiome and MAFLD, which may contribute to developing strategies for the prevention, diagnosis, and treatment of MAFLD.

## Data availability statement

The data presented in the study are deposited in the NCBI Sequence Read Archive (SRA) repository, accession number PRJNA929590 (https://www.ncbi.nlm.nih.gov/bioproject/929590, PRJNA929590).

## Ethics statement

The studies involving human participants were reviewed and approved by Ethics Committee of School and Stomatology Wenzhou Medical University. The patients/participants provided their written informed consent to participate in this study.

## Author contributions

MW, C-GN and C-YQ performed the study design, data analysis, drafting, and revising of the work. L-YY and C-CZ performed the data analysis and acquisition. Y-HP and Z-HW contributed to the study design, clinical sample collection, data analysis, drafting, revising, and final approval. All authors contributed to the article and approved the submitted version.
